# Low-Dose Rivaroxaban to Prevent Recurrences of Venous Thromboembolism in Cancer: A Real-Life Experience with a Focus on Female Patients

**DOI:** 10.3390/jcm12196427

**Published:** 2023-10-09

**Authors:** Paolo Santini, Carolina Mosoni, Alessandro D’Errico, Enrica Porceddu, Andrea Lupascu, Emanuele Valeriani, Paolo Tondi, Roberto Pola, Angelo Porfidia

**Affiliations:** 1 Thrombosis Clinic, Fondazione Policlinico Universitario A. Gemelli IRCCS, L.go A. Gemelli 8, 00168 Rome, Italy; paolo.santini@guest.policlinicogemelli.it (P.S.); carolina.mosoni@guest.policlinicogemelli.it (C.M.); alessandro.derrico@guest.policlinicogemelli.it (A.D.); andrea.lupascu@policlinicogemelli.it (A.L.); angelo.porfidia@policlinicogemelli.it (A.P.); 2 Division of Angiology, Heart and Vessel Department, Lausanne University Hospital, 1005 Lausanne, Switzerland; enrica.porceddu@chuv.ch; 3 Division of Angiology, Fondazione Policlinico Universitario A. Gemelli IRCCS, L.go A. Gemelli 8, 00168 Rome, Italy; paolo.tondi@unicatt.it; 4 Department of General Surgery and Surgical Specialty, Sapienza University of Rome, Piazzale Aldo Moro 5, 00185 Rome, Italy; emanuele.valeriani@uniroma1.it; 5 Department of Translational Medicine and Surgery, Università Cattolica del Sacro Cuore, L.go A. Vito 1, 00168 Rome, Italy

**Keywords:** anticoagulants, neoplasms, cancer, rivaroxaban, venous thromboembolism, thrombosis

## Abstract

Background: The way in which to prevent recurrent venous thromboembolism (VTE) is an unmet clinical need in cancer patients. International guidelines only provide conditional recommendations and do not specify which anticoagulant and dose should be used. In the last 2 years, we have been using low-dose rivaroxaban to prevent VTE recurrences in cancer patients. The results of this real-life experience are presented in this study. Methods: All patients had cancer and had previously completed a cycle of at least six months of full-dose anticoagulation for the treatment of a VTE index event, before receiving a prescription of low-dose rivaroxaban (10 mg once daily) for secondary prevention of VTE. Effectiveness and safety of this therapeutic regimen were evaluated in terms of VTE recurrences, major bleedings (MB), and clinically relevant non-major bleedings (CRNMB). Results: The analysis included 106 cancer patients. Their median age was 60 years (IQR 50–69). Metastatic cancer was present in 87 patients (82.1%). Six patients (5.7%) had brain metastases. Over a median follow-up time of 333 days (IQR 156–484), the incidence of VTE recurrences was 3.8% (95%CI 1.0–9.4), with a recurrence rate of 4.0 per 100 person-years (95%CI 1.1–10.2). We observed no MB (0.0%) and three CRNMB (2.8%) (95%CI 0.6–8.1). Conclusions: Low-dose rivaroxaban is potentially effective and safe in cancer patients that require prevention of recurrent VTE. Large-scale studies are needed to confirm these findings.

## 1. Background

Optimal type, dose, and duration of long-term (>6 months) anticoagulation to prevent recurrent venous thromboembolism (VTE) in cancer patients is not clear yet [1,2]. International guidelines suggest long-term anticoagulation for secondary prophylaxis (>6 months), but this recommendation is conditional and with low certainty in the evidence of effects [3]. The same guidelines admit that no data are available to prefer direct oral anticoagulants (DOACs) or low-molecular weight heparins (LMWH) as long-term anticoagulation in oncological patients and do not make any hint on which dose should be used [3].

Additional information on this topic will be soon provided by the API-CAT (APIxaban Cancer Associated Thrombosis) study, which is an ongoing randomised, double-blind, clinical trial designed to ascertain the non-inferiority of a low-dose regimen of apixaban (2.5 mg twice daily) versus a full-dose regimen of apixaban (5 mg twice daily) for the prevention of recurrent VTE in patients with active cancer who have completed at least 6 months of anticoagulant therapy for an index thrombotic event [4]. While waiting for the results of the API-CAT trial, Larsen and colleagues recently published the results of a single arm interventional clinical trial that assessed the efficacy and safety of low-dose apixaban for secondary prevention of VTE in cancer patients [5]. They studied 196 patients with active cancer who continued with low-dose apixaban 2.5 mg twice daily for another 30 months after 6 months treatment with full-dose apixaban of an index thrombotic event. They found that 14 patients (7.6%) had recurrent VTE, 6 patients (3.1%) had major bleeding (MB), and 16 patients (8.1%) had clinically relevant non-major bleeding (CRNMB). To our knowledge, these are the only data available in the literature on efficacy and safety of low-dose DOACs for the secondary prevention of VTE in cancer patients.

Since January 2020, we have been prescribing low-dose rivaroxaban (10 mg once daily) for the prevention of recurrent VTE in patients with cancer who had completed at least 6 months of full anticoagulant therapy for the treatment of a documented index thrombotic event. Here, we present the results of this real-life experience, with data on effectiveness and safety after a median follow-up of about 1 year.

## 2. Methods

Starting on January 2020, at the “Section of Internal Medicine and Thromboembolic Diseases” of the A. Gemelli University Polyclinic, Rome, Italy, we decided to prescribe low-dose rivaroxaban (10 mg once daily) to prevent VTE recurrences in cancer patients. The enrolment period lasted from January 2020 to December 2021, while the data collection period ended in October 2022. Data collection was conducted through the acquisition of follow-up visits which were carried out at the discretion of the treating physician, based on the clinical conditions of each individual patient. We have been using this therapeutic strategy in unselected oncological patients, excluding only those who had an absolute contraindication to anticoagulation, such as platelet count <25 × 10^9^/L, active or recent major bleeding, coagulation diseases with haemorrhagic propensity, and hepatic disease associated with elongation of international normalised ratio (INR). We also excluded from this therapeutic regimen patients who could not take oral therapy and those who had a contraindication to the use of DOACs, such as calculated creatinine clearance < 15 mL/min, increased level of transaminases or bilirubin, and concomitant use of strong inhibitors of CYP3A4 and P-glycoprotein. We also excluded pregnant or potentially pregnant women, female patients who were breastfeeding, and patients with life expectancy < 6 months. Also, patients with indication for continuing anticoagulation at full therapeutic dose, such as those affected by atrial fibrillation, were excluded.

Beyond these exceptions, a therapeutic regimen with low-dose rivaroxaban has been prescribed to all patients affected by active cancer, or with history of cancer (within the previous two years), who had just completed (within 7 days) at least 6 months of full-dose anticoagulant therapy with LMWHs or a DOAC for treating a documented index thrombotic event and needed long-term anticoagulant therapy for the prevention of recurrent VTE. The definition of active cancer was as established in the literature, i.e., cancer diagnosed within the previous 6 months; recurrent, regionally advanced, or metastatic cancer; cancer for which treatment had been administered within the previous 6 months; or hematologic cancer that was not in complete remission [6]. This study also includes a limited number of patients who had no active cancer but had a history of cancer within the previous 2 years. All the patients analysed in this study had documented evidence of at least 6 months of full anticoagulation for the treatment of an index thrombotic event. All anticoagulant treatments (unfractionated heparin, LMWH, DOAC, vitamin K antagonist) were allowed, also in combination or in sequence, if they had been administered at the proper therapeutic dose. All patients included in our analysis started therapy with low-dose rivaroxaban within 7 days from the end of their full anticoagulation regimen. The patients analysed in our study had various types of index thromboembolic events, such as deep vein thrombosis (DVT) of the lower limbs, pulmonary embolism (PE), and upper extremities DVT (UEDVT), often associated with the presence of a central venous catheter (CVC).

We have been following these patients after prescription of low-dose rivaroxaban in order to assess effectiveness and safety of this therapeutic strategy. To determine effectiveness, we calculated incidence and rate of objectively verified recurrent VTE. To determine safety, we assessed the incidence of MBs and CRNMBs, defined according to the criteria of the International Society of Thrombosis and Haemostasis [7].

Baseline characteristics of the population are described by means and standard deviation, or by median and interquartile range, in the cases of continuous variables with normal and non-normal distribution, respectively. Categorical variables are described by percentage frequency. Outcomes are described as cumulative incidences and incidence rates. Cumulative incidence is calculated with 95% confidence intervals (CIs) using the binomial exact distribution. Incidence rates are reported as percent per person-years with 95% CI based on the Poisson distribution. The outcomes of efficacy and safety are calculated considering a time of follow-up which starts from the prescription of low-dose rivaroxaban to the first censoring event (i.e., death, recurrence of venous thromboembolism, major bleeding, suspension, or replacement of the low-dose rivaroxaban therapy with another anticoagulant drug by clinical decision of the attending physician). Statistical analysis was performed using STATA^®^ software (version 17.10 BE).

The study was conducted in accordance with the Declaration of Helsinki. The analyses have been carried out as part of protocol number 49904/18, approved by the Ethics Committee of the Fondazione Policlinico Universitario A. Gemelli IRCCS.

## 3. Results

Our analysis included 106 patients. Their clinical and demographic characteristics are presented in Table 1. Briefly, the median age was 60 (IQR 50–69), and the male/female ratio was 6/100. Active cancer was present in 94 patients (88.7%), while 12 patients (11.3%) had a history of cancer within the previous 2 years. The most frequent cancer types were ovarian (40.6%), breast (29.2%), endometrial (16.0%), cervical (6.6%), and colorectal (4.7%). Patients affected by gastric cancer, lung cancer, prostate cancer, cholangiocarcinoma, ampullary carcinoma, tonsil carcinoma, and haematologic neoplasm were also present. A total of 87 patients (82.1%) had metastatic cancer. The index VTE event was a thrombosis in a usual site (deep veins of the lower limbs and/or pulmonary arteries) in 60 patients (56.6%) and a UEDVT in 46 patients (43.4%), almost always associated with the presence of a CVC (44 patients). The median follow-up time on low dose rivaroxaban was 333 days (IQR 156–484). Loss to follow-up occurred in 3 patients (2.8%).

The effectiveness and safety outcomes of the study are presented in Table 2. Among the 106 patients treated with low-dose rivaroxaban, we registered 4 VTE recurrences, with an incidence of 3.8% (95%CI 1.0–9.4) and a recurrence rate of 4.0 per 100 person-years (95%CI 1.1–10.2). The four recurrences were all proximal DVTs of the lower limbs. In terms of safety, we did not observe any MB (incidence 0.0%). There were three CRNMBs, with an incidence of 2.8% (95%CI 0.6–8.1). The three CRNMBs consisted of one gastrointestinal bleeding, one genital bleeding, and one haemothorax.

Since our cohort included a relevant proportion of patients in whom the index thrombotic event was a UEDVT (43.4%), we decided to separately assess effectiveness and safety outcomes in subjects who had a thrombosis in a usual site and those who had a thrombosis in the upper extremities. We found that all thromboembolic recurrences occurred in the group of patients with an index event of VTE in usual site. Therefore, in this population, the VTE recurrence on low-dose rivaroxaban therapy occurred with a cumulative incidence of 6.7% (95%CI 1.9–16.2) and a rate of 7.5 per 100 person-years (95% CI 2.1–19.3). On the other hand, incidence of recurrence was 0.0% in the group of patients who had the index thrombotic event in the upper limb.

The clinical characteristics of the four patients who presented VTE recurrences are presented in Table 3. Of note, two of these patients had an extrinsic vascular compression by the tumoral mass in the groin region on the side of the DVT recurrence.

## 4. Discussion

Upon publication of the AMPLIFY-EXT and EINSTEIN CHOICE trials, extended therapy with low doses of DOACs (apixaban and rivaroxaban) has become a common strategy for preventing VTE recurrences. However, it is not known whether such therapeutic regimens are effective and safe in cancer patients, due to a lack of representation of this population in these trials. In detail, the numbers of cancer patients in the low-dose arms of the AMPILFY-EXT and EINSTEIN CHOICE trial were of 15 and 27, respectively [8,9]. Therefore, secondary prevention of VTE in cancer patients unanimously remains an unmet clinical need [10]. Our study provides novel real-life information on the possible ability of low-dose rivaroxaban to prevent VTE recurrences in patients with cancer. Over a median follow-up of about 1 year, the incidence of VTE recurrence was 3.8% in the whole study cohort (with a recurrence rate of 4.0 per 100 person-years) and 6.7% in the subgroup of patient who had the VTE index event in a usual site (with a recurrence rate of 7.5 per 100 person-years). Interestingly, no patient with UEDVT experienced the occurrence of a thromboembolic recurrence. We should ask ourselves whether these rates of recurrence are higher or lower, compared either to no treatment, treatment with other low-dose anticoagulants, and treatment with full-dose anticoagulation. Regarding the comparison with no treatment, it has been estimated that, after stopping anticoagulation, patients with cancer are two times more likely to have a recurrent VTE event compared to non-cancer patients with transient risk factors [11]. It has also been reported that the cumulative recurrent VTE incidence is 13.5% at 1 year in cancer patients who stop anticoagulation [12]. Based on this, we might state that the incidence of VTE recurrence that we have registered during extended treatment with low-dose rivaroxaban is lower than that reported in the literature in patients with cancer that stop anticoagulation. Regarding the comparison with other low-dose anticoagulation strategies, the only possible reference is the aforementioned study recently published by Larsen and colleagues [5], in which a recurrence rate of 8.3 per 100 person-years was observed during the first 12 months of therapy with low-dose apixaban. Such recurrence rate is higher than what we observed using low dose rivaroxaban. Regarding the comparison with full-dose anticoagulation, if we look at the DALTECAN study, a clinical trial that evaluated anticoagulation with dalteparin beyond 6 months in patients with cancer-associated VTE, we may see that VTE recurred in 4.1% of patients over a follow-up period of 6 months [13]. On the other hand, if we look at the TiCAT study, a prospective, open, single-arm, multicentre study in patients with cancer-associated thrombosis treated with tinzaparin long term (beyond 6 months), VTE recurred in 1.1% of treated patients between the seventh and the twelfth months [14]. These findings suggest that full-dose anticoagulation might be more efficacious than low-dose strategies to prevent VTE recurrences in cancer patients, but direct comparisons between these studies cannot be made, due to the different design, sample size, time of observation, and anticoagulant used.

In terms of safety, the incidence of MB that we registered in our cohort was 0.0%, while the incidence of CRNMB was 2.8%, with a little difference among subject who had the index thrombotic event in a usual site (3.3%) and in an unusual site (2.2%). This is an interesting finding, considering that Larsen and colleagues reported a non-irrelevant number of MBs in their study with low-dose apixaban (3 MBs in 196 patients in the first 6 months of treatment and 1 MB in 132 patients in the following 6 months) [5]. It is also interesting to note that in the TiCAT study, 12 MBs were reported on a total of 247 patients treated with full-dose tinzaparin (with an incidence of 4.9%) [14]. A similar incidence of MB was seen in the DALTECAN study, among patients treated with full-dose dalteparin [13]. Taken together, these data suggest that low-dose rivaroxaban is a safe therapeutic strategy in cancer patients that require secondary prevention for VTE.

Besides the therapeutic regimen used, our study has additional interesting peculiarities. First, the investigated population mainly consisted of women (94.3%) affected by either gynaecologic or breast cancer (92.4%). Therefore, this study provides unique gender-specific information, with a specific view on female cancers. Another peculiarity of our study is that it included a considerable number of patients with a VTE index event that occurred in the upper limb (43.4%), almost always in association with the presence of a CVC. According to a recent analysis that has used the RIETE registry, the incidences of recurrent VTE and PE among patients with catheter-related UEDVT are 3.2 and 3.8 per 100 patient-years, respectively [15]. A high recurrence rate in these patients has been found also in a systematic review by Thiyagarajah and colleagues [16]. Moreover, in a recent meta-analysis, patients with catheter-related UEDVT showed a VTE recurrence incidence over 13 months of 3% (95%CI: 2–4) and an incidence of major bleeding of 4% (95% CI: 3–7), over 13 months [17].

In this scenario, it is interesting that we did not observe any thrombotic recurrence in the 46 cancer patients who were on secondary prophylaxis with low-dose rivaroxaban after a UEDVT.

This study has the limitation of being a real-life experience, with susceptibility to multiple sources of bias. In addition, the fact that women and female cancers were over-represented, if on one side might be an interesting peculiarity, on the other side might limit the applicability of our findings to the general population of patients with cancer. Accordingly, due to lack of adequate representation of male patients, conclusions can only be applied to female patients. Another potential limitation is that we did not include only patients with active cancer, but also a certain number of subjects with history of cancer within the previous two years (11.3%). However, this is not inappropriate, since also the HOKUSAI-VTE CANCER and the CARAVAGGIO trials included patients with cancer diagnosed within 2 years before enrolment [18,19]. One could reasonably argue that patients with active cancer and those with a history of cancer do not carry the same risks of recurrent VTE and bleeding [20]. However, there are subgroup analyses of the EINSTEIN and HOKUSAI studies that found similar rates of recurrent VTE and bleeding between these categories [21,22]. Nonetheless, whether the inclusion of patients with a history of cancer might have introduced some bias by decreasing the rates of recurrent VTE and bleeding remains an unanswered issue.

In conclusion, our real-life data provide novel information on the use of low-dose rivaroxaban to prevent recurrent VTE in cancer patients. The rate of recurrence associated with this therapeutic strategy is similar to what has been recently reported in subjects treated with low-dose apixaban, while the incidence of bleeding appears to be low. Large-scale studies are needed to confirm these findings.

## Figures and Tables

**Table 1:** Demographic and clinical characteristics of the study population.

Characteristics	Total (*n* = 106)
Male/female ratio	6/100
Median age, yrs (IQR)	60 (50–69)
Cancer activity, n (%)	
- Active cancer	94 (88.7)
- History of cancer	12 (11.3)
Active oncological treatment	89 (84.0)
- Chemotherapy	63 (59.4)
- Immunotherapy	26 (24.5)
- Hormonal therapy	29 (27.3)
- Antiangiogenic therapy	16 (15.1)
- Radiotherapy	18 (17.0)
Cancer stage, n (%)	
- Localised	25 (23.6)
- Metastatic (without evidence of brain metastasis)	81 (76.4)
- Metastatic (with brain metastasis)	6 (5.7)
Primary cancer site, n (%)	
- Breast	31 (29.2)
- Ovarian	43 (40.6)
- Endometrial	17 (16.0)
- Cervical	7 (6.6)
- Colorectal	5 (4.7)
- Stomach	1 (0.9)
- Lung	1 (0.9)
- Prostate	1 (0.9)
- Bile duct	1 (0.9)
- Hepatopancreatic ampulla	1 (0.9)
- Tonsil	1 (0.9)
- Haematologic neoplasm	1 (0.9)
- Synchronous	4 (3.8)
Type of VTE index event, n (%)	
- Usual site VTE	60 (56.6)
- Unusual site VTE	46 (43.4)
VTE index event site, n (%)	
- Pulmonary embolism	39 (36.8)
- Deep vein thrombosis of the lower limbs	21 (19.8)
- Deep vein thrombosis of the upper limb (catheter-related)	43 (40.6)
- Deep vein thrombosis of the upper limb (non-catheter-related)	3 (2.8)
Previous history of VTE, n (%)	9 (8.5)
Comorbidities, n (%)	
- Hypertension	50 (47.2)
- Dyslipidaemia	21 (19.8)
- Diabetes	10 (9.4)
- Inflammatory bowel disease	3 (2.8)
- Liver cirrhosis	1 (0.9)
Creatinine clearance < 50 mL/min, n (%)	26 (24.7)
Median follow-up time on low-dose rivaroxaban, days (IQR)	333 (156–484)

**Table 2:** Effectiveness and safety outcomes.

Outcomes	Whole Cohort	Patients with Index Event in Usual Site	Patients with Index Event in Upper Limbs
Effectiveness			
VTE recurrences, n/total (%)	4/106 (3.8)	4/60 (6.6)	0/46 (0.0)
VTE recurrence rate, n per 100 persons per year	4.0	7.5	0.0
Type of VTE recurrence			
- PE, n/total (%)	0/4 (0.0)	0/4 (0.0)	0/4 (0.0)
- Proximal DVT of lower limbs, n/total (%)	4/4 (100.0)	4/4 (100.0)	0/4 (0.0)
Safety			
MB, n/total (%)	0.0	0.0	0.0
CRNMB, n/total (%)	3/106 (2.8)	2/60 (3.3)	1/46 (2.2)
Site of CRNMB			
- Gastrointestinal, n/total (%)	1/3 (33.3)	1/2 (50.0)	0/1 (0.0)
- Genital, n/total (%)	1/3 (33.3)	1/2 (50.0)	0/1 (0.0)
- Haemothorax, n/total (%)	1/3 (33.3)	0/2 (0.0)	1/1 (100.0)
All-cause mortality rate, n per 100 persons per year	11.0	13.2	8.5

**Table 3:** Description of VTE recurrences.

	Patient #1	Patient #2	Patient #3	Patient #4
Age	76	56	66	57
Sex	Female	Female	Female	Male
BMI	35.3	30.9	29.4	23.7
Cancer activity	Active cancer	Active cancer	Active cancer	History of cancer
Cancer site	Ovarian	Endometrial	Ovarian	Tonsil
Cancer stage	Metastatic	Metastatic	Metastatic	Localised
Active oncological treatment	Yes	Yes	Yes	No
VTE index event	PE and proximal DVT of lower limbs (May 2021)	PE (August 2021)	PE and proximal DVT of lower limbs (September 2021)	Proximal DVT (February 2022)
Previous anticoagulant regimen (days)	Enoxaparin 6000 U.I. twice daily (209)	Enoxaparin 6000 U.I. twice daily (52) followed by edoxaban 60 mg once daily (128)	Enoxaparin 6000 U.I. twice daily (29) followed by apixaban 5 mg twice daily (165)	Enoxaparin 6000 UI twice daily (71) followed by edoxaban 60 mg once daily (116)
Days from VTE to dose reduction	215	180	194	187
VTE recurrence	Proximal DVT	Proximal DVT	Proximal DVT	Proximal DVT
Days from dose reduction to VTE recurrence	292	130	104	248
Extrinsic vascular compression	Yes	No	Yes	No

## Data Availability

The data presented in this study are available on request from the corresponding author. The data are not publicly available due to ethical issues.
